# Resilient Privacy Preservation Through a Presumed Secrecy Mechanism for Mobility and Localization in Intelligent Transportation Systems

**DOI:** 10.3390/s25010115

**Published:** 2024-12-27

**Authors:** Meshari D. Alanazi, Mohammed Albekairi, Ghulam Abbas, Turki M. Alanazi, Khaled Kaaniche, Gehan Elsayed, Paolo Mercorelli

**Affiliations:** 1 Department of Electrical Engineering, College of Engineering, Jouf University, Sakakah 72388, Saudi Arabia; 2 School of Electrical Engineering, Southeast University, Nanjing 210096, China; 3 Department of Electrical Engineering, College of Engineering, University of Hafr Al Batin, Hafr Al Batin 39524, Saudi Arabia; 4 Department of Interior Design, College of Engineering, Jouf University, Sakakah 72388, Saudi Arabia; 5 Institute for Production Technology and Systems (IPTS), Leuphana Universität Lüneburg, 21335 Lüneburg, Germany

**Keywords:** forward secrecy, intelligent transportation, smart cities, transfer learning, machine learning

## Abstract

An intelligent transportation system (ITS) offers commercial and personal movement through the smart city (SC) communication paradigms with hassle-free information sharing. ITS designs and architectures have improved via information and communication technologies in recent years. The information shared through the communication medium in SCs is exposed to adversary risk, resulting in privacy issues. Privacy issues impact the contingent mobility and localization of the ITS path. This paper introduces a novel resilient privacy preserving (RPP) method through presumed secrecy (PS) to provide a robust privacy measure. The privacy of the progressive communication sessions is preserved based on the previous security depletion levels. The interruptions in traffic data-related communication sessions are recurrently identified, and re-handoffs are recommended with dodged transfer learning. The empirical results indicate a 25% reduction in computational overhead and a 30% enhancement in privacy protection over conventional methods, demonstrating the model’s efficacy in secure ITS communication. Compared with existing methods, the proposed approach decreases security depletion rates by 15% across varying traffic densities, underscoring ITS resilience in high-interaction scenarios.

## 1. Introduction

Data and communication privacy are critical in intelligent transportation systems (ITSs), which handle sensitive information such as vehicle positions and personal details. The connectivity between automobiles and infrastructure raises the possibility of data leaks [1]. Unauthorized access can result in catastrophic consequences, such as identity theft and data manipulation. Protecting communication privacy is critical for maintaining user safety and confidence [2]. Encryption and anonymization techniques contribute to the security of sensitive data. The dynamic nature of these systems necessitates ongoing attention to privacy concerns. A well-secured communication channel increases security and public trust [3,4]. Effective privacy protections keep hostile actors from exploiting transportation data. Strong privacy protection techniques are required as digital communication merges with transportation [5]. Data integrity and privacy are critical for efficient operations in smart city infrastructures. Addressing privacy concerns safeguards consumers and enhances transport systems. Cities that promote privacy can provide a safer environment for their citizens. A dedication to communication privacy is critical to the future of an ITS [6,7].

Session authentication is critical for secure communication in an ITS because it confirms the identities of all parties involved. Such a method prevents unauthorized access and ensures that only legitimate users share sensitive transportation data [8]. Variable key exchanges establish secure connections, lowering the danger of interception or manipulation. Regular re-authentication improves communication security, particularly in real-time traffic systems requiring consistent data flow [9]. Lightweight cryptographic approaches ensure that security measures do not reduce data transmission efficiency. These techniques are critical for preserving the speed and dependability of an ITS. Authentication protocols detect and respond to potential security risks, resulting in a safe environment for data exchange [10,11]. Proper session authentication prevents unauthorized access and ensures communication integrity. Such security is critical for establishing trust in intelligent transportation technologies [12]. Effective session authentication protects sensitive information and increases user confidence. To satisfy changing security requirements, authentication systems must be continuously improved. New strategies are always being developed to successfully address difficulties [13].

Machine learning (ML) techniques are essential for ensuring privacy in an ITS. These allow for the real-time identification and mitigation of potential security issues. These algorithms can find trends in massive datasets from transportation systems that indicate weaknesses or assaults [14]. ML models improve security by predicting and preventing future intrusions. Automated detection and response systems that use ML eliminate the need for manual monitoring [15]. Such technologies evaluate the degree of security issues, enabling more precise responses. ML improves encryption algorithms and detects holes in existing security protocols [16]. The capacity to handle huge amounts of data enables an ITS to avoid new hazards. Using ML improves privacy and overall security in transportation networks [17]. Such integration results in more efficient operations and improved resource allocation. As technology improves, ML’s significance in ensuring security will become more important [17]. A bi-level planning model was proposed in [18] to locate and size Multi-Functional Charging Station (MFCS) which can recharge Battery Electric Vehicle (BEV), Hydrogen Fuel-Cell Vehicle (HFCV), and Natural Gas Vehicle (NGV) at the same time in a medium-sized city with different functional areas. The proactive strategy allows organizations to respond quickly to anticipate difficulties. ML and intelligent transportation will collaborate to create future developments and improve system performance [19].

Several limitations exist to the efficiency of the existing privacy-preserving techniques for mobility and localization in intelligent transportation systems (ITSs). For real-time use, a reasonable compromise between privacy and location accuracy is lacking in many existing solutions. Even with encryption and obfuscation, they are still susceptible to complex attacks like inference and de-anonymization attacks that utilize machine learning to reveal user identities. In addition, these methods are not always scalable; thus, they cannot keep up with the increasing volume of cars and gadgets in large-scale ITS schemes. However, as most of these techniques fail to work with advanced localization algorithms, including those based on probabilistic or deep learning, the system performance is less than ideal. Due to these difficulties, a more robust, scalable, and integration-friendly strategy is required to guarantee strong privacy in ever-changing ITS environments.

The contributions are as follows:

- A resilient privacy preserving (RPP) mechanism with adaptive security, which proposes enhanced privacy in intelligent transportation systems (ITSs).
- A dodged transfer learning approach is developed for proactive breach detection and optimized re-handoff recommendations.
- A mutual lightweight key exchange protocol is introduced to reduce adversarial access during vehicle handoffs.
- Comparative analysis is conducted, which demonstrates the improved security and computational efficiency of the existing method.

The remainder of the paper is as follows: Section 3 discusses the recent literature review on the proposed topic. Section 3 describes in detail the proposed methodology and the results obtained. The discussion is carried out in Section 4. The conclusion of the paper is drawn in Section 5.

## 2. Related Works

Qureshi et al. [20] developed a blockchain (BC)-based privacy-preserving authentication (BPPAU) model for intelligent transportation systems (ITSs). Data storage and processing management are analyzed to identify the threats that cause data leakage and data losses. The BPPAU model provides a secure solution to complex ITS application threats. The developed BPPAU model improves the performance and security range of an ITS. Chen et al. [21] introduced a differential privacy (DP)-based privacy-preserving data publishing (PPDP) algorithm for smart transportation. A query probability model is employed here to identify the errors while performing preserving tasks. The introduced algorithm analyzes the impact of smart card trajectory data, producing a feasible set of information for PP services. The introduced algorithm maximizes the computational efficiency range of the systems. Gao et al. [22] proposed a location privacy-oriented task offloading (PPO2) method for an ITS. A deep reinforcement learning (DRL) algorithm is implemented to provide necessary planning services. The proposed method is used to identify the offloading problems that cause issues with the PP service. The DRL algorithm minimizes the risk factor, elevating the systems’ efficiency. The proposed PPO2 method reduces the computational cost and privacy data loss rate in an ITS.

Li et al. [23] developed a PP-distributed transfer learning protocol for an ITS. The protocol solves the system’s scarcity level, elevating the functional capabilities. The developed protocol provides effective PP services to ensure the safety and security range of users’ data during data transmission and sharing processes. The developed protocol enlarges the efficiency and effectiveness of ITS applications. Saleem et al. [24] designed a conditional-privacy access control protocol for intelligent customer-centric communication in VANET. A trusted authority (TA) is employed here to tackle the issues that cause risks and threats to the user’s data. The TA also analyzes the data’s security range, eliminating the process’s compactional cost. Experimental results show that the designed protocol achieves high accuracy and efficiency in providing privacy services. Din et al. [25] introduced a context-aware cognitive memory trust management system (CACMTM) model for an ITS. The introduced model is used to provide trustworthy communication services to the users of ITS applications. The context-aware cognitive memory trust management system (CACMTM) model identifies the demands and requests that the users make. The identified demands are analyzed to provide optimal services. The introduced model enlarges the safety and privacy ratio of user data.

Zhu et al. [26] developed a poisoning attack method using federated learning (FL) with a perturbation coefficient multiplied by maximum value (PMM) for ITS applications. The developed method is used to identify the hierarchical poisoning attacks and defenses that occur during data transmission. The method also provides specific PP services to secure the data leakage range in an ITS. The developed method maximizes the effectiveness level of the systems. Qu et al. [27] proposed a new personalized FL (PFL)-based information fusion method for privacy protection in an ITS. The PFL is used here to analyze the data nodes which contain users’ personal information. It also eliminates the unwanted noise which is presented in the data. The method evaluates the parameters and provides feasible PP services to the data. Compared with others, the proposed method enhances the accuracy of privacy protection in an ITS applications. The methods and findings from [28,29,30,31,32,33,34,35,36,37] are summarized in Table 1.

Developing an ITS in smart cities is familiar in areas where road side units (RSU) aid autonomous vehicle transmission. The role of this RSU is to sense the traffic on the road and transmit it to the nearby vehicle that reaches the same traffic flow path. This communication facilitates information exchange from the RSU to avoid unnecessary traffic. It includes mutual key authentication with secure traffic data exchange between the vehicles. Using a handoff mechanism, the RSU is responsible for sharing the data with the nearby vehicle. This mechanism is used to identify the interlinked vehicle and the ITS data that are to be exchanged with each other. Analyzing this traffic management phase, the security-related process is executed using encryption and decryption. Accuracy, privacy, and scalability are challenges for traditional approaches when dealing with the massive amounts of real-time data produced by infrastructure, connected vehicles, and sensors. Machine learning algorithms effectively, efficiently and accurately predict outcomes from massive datasets containing various variables. Machine learning (ML) improves the balance between privacy and system efficiency, allows for reliable localization even in unpredictable environments, and adjusts to new security risks. The suggested system can adapt to changing network circumstances, complex privacy assaults, and the need to integrate with other ITS components due to ML.

## 3. Proposed Resilient Privacy Preservation (RPP) Through Presumed Secrecy (PS)

The proposed work focuses on mutual key exchange by reducing adversary control and evaluating the security level of data sharing. In this processing step, smart cities allow autonomous vehicles to transmit among each other, and the analysis is used to observe the key generation, which is said to be a handoff. The handoff works remotely on two vehicles; based on the user, access is given on one device, paused, and then continues on toward other devices with the required security. From this examination phase, the handoff key is used to observe the interrelated road traffic management issues. This work mainly focuses on privacy-related issues handled using handoff- and recommendation-based techniques. As discussed in the section below, the proposed technique is one of transfer learning for a dodged mechanism.

In the execution step, traffic management is used to decrease the traffic flow and reduce congestion. This is undertaken using transfer learning with the existing knowledge of autonomous vehicles and roads; knowledge which provides the relevant information needed to avoid traffic. This step measures the security level without any adversary accessing the data from the vehicle. The initial step is to discuss the challenges faced when there are interrelated traffic management systems, which are observed in the equation below.

(1) $$Yi^{\prime }\left(T_{f},M_{g}\right)=\sum_{h_{0}}^{h_{1}}\left(M_{s}+f_{w}\right)*n^{\prime }+F_{d}*pr_{0}$$

The above Equation (1) represents the traffic management analysis for the traffic flow. The analysis is described as ${Yi}^{\prime }$, $T_{f}$ is the traffic and $M_{g}$ is the management, vehicle is $h_{0}$, n number of vehicles is $h_{n}$, and the information is $n^{\prime }$. The smart cities are labeled as $M_{s}$, identification is $F_{d}$, and the transportation is $pr_{0}$. From these derivatives, the transportation of information is carried out $pr_{0}(n^{\prime })\to h_{0}$.

**Definition** **1.** *Consider* $Yi^{\prime }\left(h_{n}\right)*n^{\prime }>f_{w}$ *, this refers to the higher traffic flow on the road. So, to avoid this* $n^{\prime }\left(pr_{0}\right)*M_{s}+R^{\prime }$*,* $R^{\prime }$ *is the RSU, where* $R^{\prime }\in M_{s}\left(T_{f}\right)+h_{n}$*. This observation step illustrates the data exchange and the condition is* $x_{h}\left(h_{0}\to h_{1}\right)*pr_{0}+T_{f}<f_{w}$.

$$h_{n}=n^{\prime }\left(f_{w}+F_{d}\right)*M_{s}\left(T_{f}\right)-pr_{0}$$

$${F_{d}}^{2}=Yi^{\prime }+\left\{\left(R^{\prime }+x_{h}\right)\to h_{n}\right\}$$

$$T_{f}\cup R^{\prime }=h_{0}+\left\{\left(pr_{0}\vee F_{d}\right),(x_{h}\wedge h_{1})\right\}$$

$$T_{f}\cap R^{\prime }=h_{0}+\left\{\left(pr_{0}\wedge F_{d}\right),(x_{h}\vee h_{1})\right\}$$

These are used to examine vehicle transportation and the exchange of information, and are symbolized as $x_{h}$, with $h_{1}$ as the nearby vehicle. The union of $T_{f}\cup R^{\prime }$ for $\left\{\left(pr_{0}\vee F_{d}\right),(x_{h}\wedge h_{1})\right\}$, whether the data exchange between vehicles is achieved or not, is represented in union data, whereas the transportation of data and identification is always carried out. The same case inversely acts on the interaction of $T_{f}\cap R^{\prime }$, as the condition delivers $\left\{\left(pr_{0}\wedge F_{d}\right),(x_{h}\vee h_{1})\right\}$. The conditions work for the union and intersection of two vehicles, and the optimization strategy works for the smart city’s data exchange. From this, traffic management in smart cities is observed in the following Equation.

(2a) $$T_{f}\left(M_{g}\right)=\left(n_{v}-\alpha \right)+\left[R^{\prime }\left(x_{h}\right)+Yi^{\prime }\right]+f_{w}$$

This is expressed as,

(2b) $$F_{d}(h_{0},\ldots h_{n})=\left[\sqrt{n_{v}}+n^{\prime }*\prod_{I_{r}}^{\alpha }\left(pr_{0}*R^{\prime }\right)\right]*\left(x_{h}\to o_{x}\right)-pr_{0}$$

From the above Equations (2a) and (2b), traffic management plays a vital role in traffic flow. Here, the identification of traffic on the road is achieved with the use of an RSU, which provides the necessary action according to $R^{\prime }\left(F_{d}*f_{w}\right)*n_{v}$. The navigation is completed if there is no traffic, or if less traffic is detected, and is represented as $n_{v}$. Congestion occurs due to traffic on the roadside, and is described as α, with the interaction labelled as $I_{r}$. In this case, the formula is $M_{g}\left(R^{\prime }+f_{w}\right)*F_{d}\to h_{n}$. On this basis, congestion is when a security level for the traffic management system is provided. The evaluation occurs for the RSU and roadside vehicles when the smart city observes a vehicle causing congestion. The congestion is managed in the below derivative.

**Definition** **2.** *Consider the congestion rate* $\left(\alpha +T_{f}\right)*\sum_{F_{d}}\left(Yi^{\prime }+M_{g}\right)$ *, where it is denoted as* $\left(F_{d}*R^{\prime }\right)+M_{s}(h_{n})$*. This is observed for the RSU-based road traffic observation and finds the trafficless route with the navigation* $n_{v}\left(T_{f}*n^{\prime }\right)*x_{h}$.

$$Yi^{\prime }=\left(n^{\prime }+R^{\prime }\right)*\left|n_{v}-F_{d}\right|+\left\{{pr}_{0}^{2}+\left(h_{0}*f_{w}\right)\right\},\;where\;pr_{0}<h_{n}$$

This means that

$$h_{n}>f_{w}\left(n^{\prime }+x_{h}\right)*\left|F_{d}+R^{\prime }\right|,\;for\;all\;R^{\prime }\in M_{s}$$

which is similarly represented as,

$$\left\{\left({\left(R^{\prime }+M_{g}\right)}^{2}*{\left|n^{\prime }+x_{h}\right|}^{-1}\right)*\left(T_{f}+pr_{0}\right)\right\},\;for\;all\;pr_{0}\in T_{f}$$

$$\mathrm{Thus},\;0\leq h_{0}\left(pr_{0}+T_{f}\right)*n_{v}$$

$$R^{\prime }(pr_{0},I_{r})=h_{0}(M_{s},\alpha )$$

(3a) $$\left(T_{f}\right).n^{\prime \left(h_{0},pr_{0}\right)}=1\Leftrightarrow \sqrt{\left[\prod_{n_{v}}^{R^{\prime }}\left(n_{v}+M_{g}\right)\right]+\prod_{I_{r}}\left(h_{1}\to h_{n}\right)}+M_{s}$$

$$pr_{0}\left(n_{v},M_{s}\right)=x_{h}(\alpha ,n^{\prime })$$

(3b) $$pr_{0}.I_{r}=0\Rightarrow \sum_{h_{0}}^{h_{n}}\left(T_{f}+n_{v}\right)*M_{s}+\sqrt{o_{x}+\left(T_{f}*R^{\prime }\right)}$$

**Figure 1:**
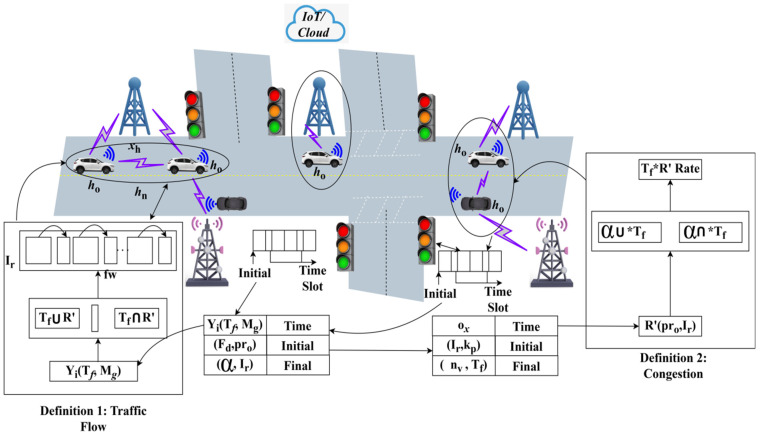
Information exchange and congestion-based traffic management.

The congestion rate is addressed in the above Equations (3a) and (3b), where the interaction is represented as $I_{r}$, $o_{x}$ and is described as communication $I_{r}\left(h_{0}\to h_{1}\right)*o_{x}\left(x_{h}\right)>0$. Navigation is used to provide traffic on the road on which the congestion has been managed during the communication $R^{\prime }+o_{x}(h_{0}\to h_{1})$. The information exchange and congestion-based traffic management are illustrated in Figure 1.

**Figure 2:**
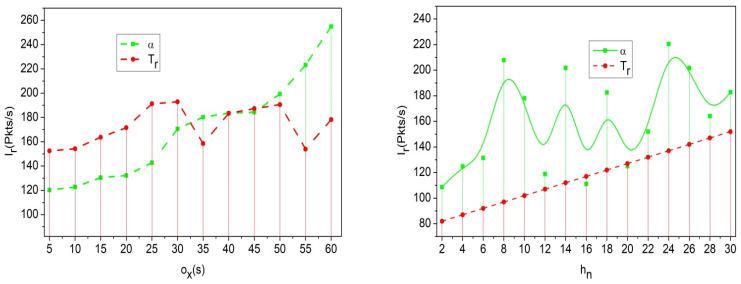
Interaction in different communication intervals.

Figure 1 illustrates the information exchange for traffic flow and congestion detection. The $I_{r}$ (initial) to final $o_{x}$ defines the rate of communication between $h_{o}\in h_{n}$ and the RSU’s. The $Y_{i}\left(T_{f},M_{g}\right)$ observed at the initial $I_{r}$ is used to extract $\left(F_{d},pr_{o}\right)$ until $\left(\alpha ,I_{r}\right)$ becomes the final $x_{h}$. In the increasing $o_{x}$, the $I_{r}$ with $k_{p}$ and $n_{v}$, while using $T_{f}$, are acknowledged to maximize congestion detection. In the detection and $x_{h}$ process, $\left(\alpha \cup T_{f}\right)$ and $\left(\alpha \cap T_{f}\right)$ are validated with $\left(T_{f}\cup R^{\prime }\right)$ and $\left(T_{f}\cap R^{\prime }\right)$ to identify the maximum difference. If the difference maximization is pursued, then $F_{d}\left(h_{o},\ldots ,h_{n}\right)$ is performed for precise information updates. Based on the above information sharing, the $I_{r}$ in any $o_{x}$ interval is evaluated, as in Figure 2.

The communication rate increases for the mutual key authentication, where transfer learning is used to analyze the secure communication with a pre-trained model $o_{x}\left(t_{p}+A_{0}\right)+h_{0}$. This evaluates the communication link among the vehicles and looks for the recommendation for handoff or re-handoff $H_{f}+L_{v}\left(f_{w}+t_{p}\right)*D_{a}$. Based on this recommendation, it uses the four parameters of transfer learning and produces a higher communication rate within the required time frame (Figure 2). The navigation is then examined and the security level is verified using a handoff. This illustrates the initialization and decision making discussed in the below section.

### 3.1. Handoff

Switching between the same access point or a different network can be either intra- or inter-technology. This is proposed in order to reduce the congestion rate between vehicle access points.

#### 3.1.1. Handoff Algorithm: Initialization

1. This is used in the initialization state of congestion to avoid unnecessary handoffs and misinterpretation, as follows: $\left(i_{m}+F_{d}\right)*\sum_{n_{v}}f_{w}>R^{\prime },\;where\;R^{\prime }\in \alpha \;$ represent the vehicle data transfer, the misinterpretation of which is $i_{m}$ and results in the below issues.
  - QoS degradation
  - Bandwidth and system load are increased or unused, leading to unused data sharing.
  - Packet losses occur due to blocking and termination during vehicle communication intervals.
2. The factors mentioned above are addressed with the vertical handoff mechanism, whereas switching to access points on a different network is undertaken as follows: $x_{h}\left(n^{\prime }\to h_{1}\right)*{\left|Yi^{\prime }+M_{s}\right|}^{2}$.

$$Int=\sum_{T_{f}}^{F_{d}}f_{w}+\left\{\left(M_{g}*R^{\prime }\right)+n_{v}\right\}-\alpha {\left|Yi^{\prime }+M_{s}\right|}^{2}$$

That computes as follows:

$$x_{h}\left(n^{\prime }\to h_{1}\right)>0,\;where\;x_{h}\in M_{g}$$

This is substituted in Equation (3a), and is formulated as follows:

(4) $$f_{w}\left(h_{0},M_{g}\right)=\sqrt{o_{x}+\left(T_{f}*R^{\prime }\right)}*i_{m}-k_{p}$$

To manage the issues mentioned above in Equation (4), an initialization is performed that addresses the packet losses and is described as $k_{p}$. The initialization is labelled as Int. This shows that the initialization of the handoff aids in the reduction of traffic using RSU.

#### 3.1.2. Handoff Algorithm: Decision Making

1. From the initialization of the handoff, the decision making is followed up as $Int\approx D^{\prime }$, where $D^{\prime }$ is the decision-making process. This refers to the higher initialization factor and resolves the handoff with misinterpretation issues, as follows: $F_{d}<i_{m}(x_{h})$.
2. The decision is taken regarding the network and surrenders the handoff as the target and provides a seamless connection between the two vehicles, as follows: $\left(H_{f}+D^{\prime }\right)+{Int}^{-1}$

$$D^{\prime }=Int+\frac{n_{v}}{h_{n}}*f_{w}+\prod_{H_{f}}x_{h}*\left(I_{r}+i_{m}\right)-R^{\prime }$$

This is represented as follows:

$$=I_{r}*\left\{\left(n_{v}+Int\right)*R^{\prime }\right\}*H_{f},\;where\;H_{f}>f_{w}$$

This is computed as in Equation (5), as follows:

(5) $$=\prod_{H_{f}}^{h_{0}}F_{d}*\left(f_{w}+T_{f}\right)*M_{g}-R^{\prime },\;for\;all\;M_{g}\in x_{h}$$

**Figure 3:**
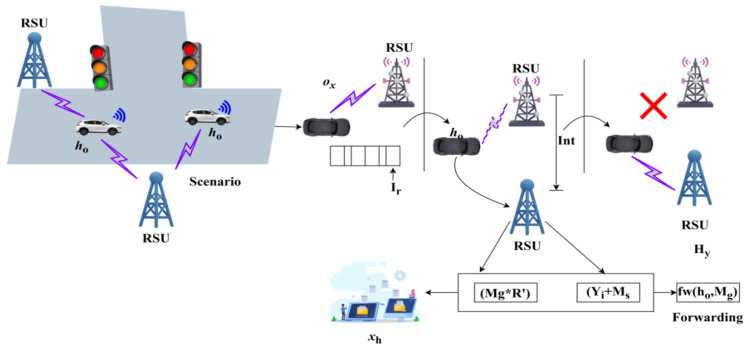
Handoff process illustration with transport management.

Thus, the decision-making is followed up for handoff, where the mutual key authentication is administered. The handoff is represented as $H_{f}$ and the interaction is $I_{r}$. The handoff process with $M_{g}$ is portrayed in Figure 3.

The Int and $H_{f}$ processes are depicted in the above Figure 3. The $I_{r}$ is interrupted by α information, such that, if $x_{h}\left(n^{\prime }\to h_{1}\right)$ (i.e., the neighbor), then Int is performed. This Int harmonizes $\left(M_{g}*R^{\prime }\right)\forall h_{0}$ and $h_{1}$ (within the range) provided $\left(Y_{i}+M_{s}\right)$ is the demanding requirement of the communicating $h_{o}\in h_{n}$. Therefore, the $\left[x_{h}\left(n^{\prime }\to h_{1}\right)>0\right]$ is a handoff-requiring case due to $k_{p}\neq 0$ outcomes. Thus, $D^{\prime }$ and $\left(f_{w}+T_{f}\right)$ in any $o_{x}$ interval is the handoff output needed to ensure (reduce) $k_{p}$ less $I_{r}$. The Presumed secrecy-based privacy is modified using different handoff authentication levels dictated by mutual key authentication, and is explained below.

### 3.2. Mutual Key Authentication

Mutual authentication is achieved using the encryption and decryption method associated with the key generation process. The private and public keys provide access to a particular vehicle and to traffic information. RSU is responsible for promptly transferring the information to the nearby vehicle without any security issues. As explained below, this ensures the private and public keys needed to access the information.

**Definition** **3.** *By examining information exchange between the vehicles, traffic management, and QoS, bandwidth and packet loss improvements are made:* $x_{h}\left(o_{x}+h_{1}\right)*\sum_{n_{v}}i_{m}\leq (T_{f},f_{w})$*.*

$$M_{k}=enc\to x_{h}\left(n^{\prime },h_{1}\right)*f_{w}+M_{g}$$

$$M_{k}=dec\leftarrow \left(\alpha -\left(f_{w}*x_{h}\right)+z_{p}-k_{p}\right)$$

The encryption and decryption are performed using the mutual key authentication $M_{k}$, which ensures higher security and privacy without adversaries.

$$=enc\left(n^{\prime }\to h_{1}\right)*R^{\prime }\left(M_{g}\right)-f_{w}$$

From this, decryption works as follows:

$$=dec\leftarrow R^{\prime }\left(M_{g}\right)+\left|n^{\prime }+z_{p}\right|*o_{x}$$

This is used as the access-based key generation method, and information sharing is followed up with higher security.

(6) $$=R^{\prime }\left(o_{x}\to h_{1}\right)*z_{p}\left(Yi^{\prime }+M_{g}\right)-f_{w}$$

From the decryption process, the congestion and packet losses are addressed optimally as in Equation (6), and are represented as $z_{p}$. The encryption and decryption are represented as enc and dec, respectively. The analysis takes place for the RSU and provides mutual authentication through encryption. The initial stage is encryption, where the data are digitally modified, and decryption is used to extract the data. This concurrent process utilizes the handoff consolidated by mutual authentication. On this basis, the recommendation for the handoff is discussed in the dodged technique and is equated below.

(7) $$D_{a}=D^{\prime }+\left(enc*T_{f}\right)+f_{w}*\prod_{Yi\prime }\left(k_{p}-\alpha \right)$$

**Figure 4:**
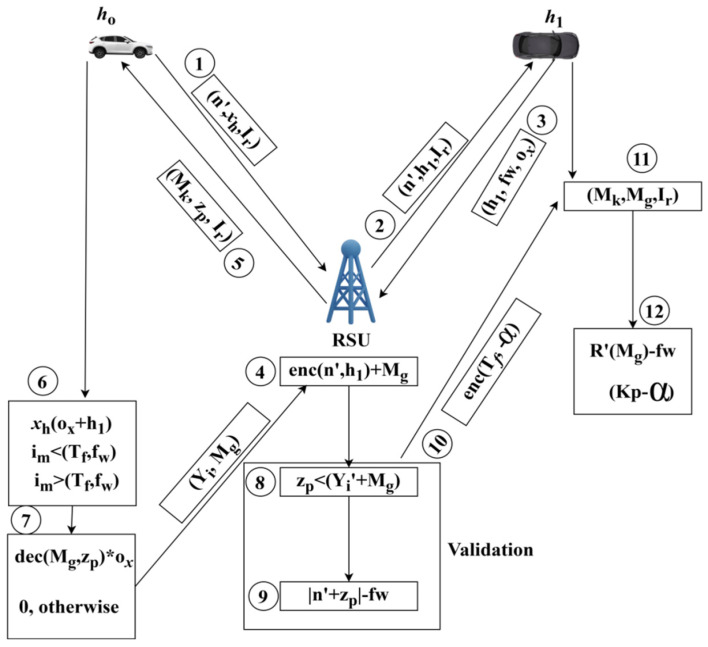
Mutual-key-based authentication illustration.

The recommendation is represented as $D_{a}$ in Equation (7), based on transfer learning, the analysis is undertaken for one vehicle and shares the information with a higher handoff that relies on security. The privacy of the progressive communication sessions is preserved based on the previous security depletion levels. Mutual-key-based authentication is illustrated in Figure 4.

**Figure 5:**
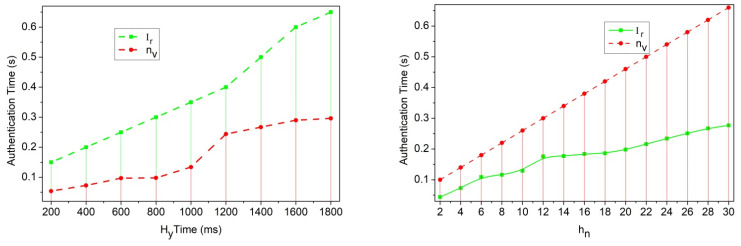
Authentication time analysis for different variants.

For the dodged $H_{f}$ process illustrated in Figure 3, the sequence of $M_{k}$-based authentication for $\left(h_{0},h_{1}\right)$ privacy in $I_{r}$ interval is represented in Figure 4. The numbered sequences in the above figure follow the authentication between two neighboring vehicles experiencing $H_{f}$. Depending on the various $\left(Y^{\prime },M_{g}\right)$ instances, the $\left[enc\left(n^{\prime },h_{1}\right)+M_{g}\right]$ (for $h_{1}\to h_{0}$) and $\left[enc\left(T_{f},-\alpha \right)\right]$ (for $h_{0}\to h_{1}$) is implied. Unke the encryption process, the decryption is consistent for $z_{p}\left(Y_{i}+M_{g}\right)$ for $f_{w}$ and $T_{f}$ concurrently. The RSU is responsible for verifying these sequences [sequences (8) and (9)] to reduce the authentication time. Therefore, $\left[dec\left[\alpha -\left(f_{w}*x_{h}\right)\right]\forall \left(z_{p}-k_{p}\right)>0\right]$ is responsible for balancing $\left(M_{k},M_{g},\;and\;I_{r}\right)$ under various $o_{x}$ intervals (Figure 4). In Figure 5, the authentication time metric is analyzed using the different related variants and functions experienced.

The authentication time is reduced among the vehicles and communicates with security. The security level is maintained based on the encryption and decryption of data, where it defines the traffic on the road and provides the data exchange according to $o_{x}\left(M_{g}+L_{v}\right)*n^{\prime }$. On this basis, transportation for the data between the vehicles is achieved on a fixed time slot and so refers to the authentication time $pr_{0}*\left(enc+H_{f}\right)+M_{k}$. This is balanced between the vehicles, and security is processed to reduce the congestion and packet losses (Figure 5). The interruptions in traffic data-related communication sessions are recurrently identified, and re-handoffs are recommended with dodged transfer learning after this transfer learning is used to identify data-related communication, with recommendations that include the handoff, as in Equation (8).

$$F_{d}=n_{v}+\left\|D_{a}*D^{\prime }\right\|-i_{m},\;where\;D_{a}\in H_{f}$$

$$n^{\prime }=T_{f}\left(M_{g}*R^{\prime }\right),\;H_{f}$$

$$x_{h}=h_{0}*\left|\alpha +M_{s}\right|-pr_{0}$$

(8) $$i_{m}=z_{p}*\sum_{D_{a}}M_{g}+(Yi^{\prime }*n_{v})$$

The identification occurs regarding the recommendation for communication followed up with the handoff. The mutual authentication process is described below:

1. The initial step discussed here is encryption, where the information is sent to the security, $x_{h}(n^{\prime })\to h_{n}$. The evaluation takes place by deciding the optimal solution among the vehicle information-sharing, $h_{0}\to h_{1}(H_{f}*f_{w})$. Based on the traffic flow, the data exchange is undertaken as follows: $x_{h}(f_{w}*enc)\to o_{x}(h_{1})$.

Examining information exchange between the vehicles relates to traffic management and addresses challenges such as QoS, bandwidth, and packet losses, $x_{h}\left(o_{x}+h_{1}\right)*\sum_{n_{v}}i_{m}\leq (T_{f},f_{w})$.

2.
$M_{k}=enc\to x_{h}\left(n^{\prime },h_{1}\right)*f_{w}+M_{g}$

3.
$M_{k}=dec\leftarrow \left(\alpha -\left(f_{w}*x_{h}\right)+z_{p}-k_{p}\right)$

The encryption and decryption are examined for the mutual key authentication $M_{k}$ where it relates to higher security and privacy without adversaries.

$$=ency\left(n^{\prime }\to h_{1}\right)*R^{\prime }\left(M_{g}\right)-f_{w}$$

From this, decryption works by substitution with step 3 and is computed as follows:

$$=dec\leftarrow R^{\prime }\left(M_{g}\right)+\left|n^{\prime }+z_{p}\right|*o_{x}$$

This is used as the access-based key generation method, and information sharing is followed up with a higher security mechanism. This is observed by integrating point 4 and decryption, defined by $R^{\prime }\left(o_{x}\to h_{1}\right)*z_{p}\left(Yi^{\prime }+M_{g}\right)-f_{w}$.

**Figure 6:**
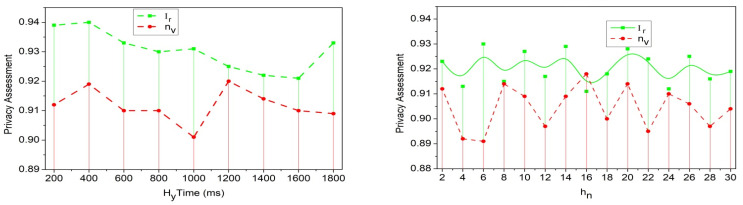
Privacy assessment rate.

From the decryption process, the congestion and packet losses are addressed in an optimization manner, represented as $z_{p}$. The encryption and decryption are represented as enc and dec, respectively. The analysis takes place for the RSU and provides mutual authentication as encryption. The initial stage is encryption, where the data are encrypted, and the decryption is used to open the data; this uses the handoff mechanism for mutual authentication. The privacy assessment rate is analyzed using the representations shown in Figure 6.

The privacy assessment increases if traffic flow is detected on the road studied by an RSU. The RSU is responsible for securely forwarding the information to the nearby vehicle, $x_{h}\to h_{0}\left(o_{x}*pr_{0}\right)-H_{f}$. The handoff works according to privacy and ensures security among end-to-end vehicles, $H_{f}\left(h_{0}\to h_{1}\right)+z_{p}(n_{v})$. This observes the traffic flow on the road and navigates the vehicle to reduce the traffic. The reduction in traffic augments privacy assessment improvements in the proposed method (Figure 6). This execution evaluates the security-based dodged technique, which relies on transfer learning and is discussed in the following section.

### 3.3. Process of Dodged Transfer Learning

The neighbor information is fetched by the second vehicle supervised by the pre-trained model. The features and starting point of the vehicles are detected using a handoff, which provides security between the vehicles. This uses four parameters to define the congestion and packet losses, which are evaluated in the equation below.

Pre-trained and base model: the transfer learning performances are based on the pre-trained model, which identifies the vehicle’s and road unit’s common features and patterns. This information is stored in the database for future reference. The base model refers to the pre-trained layer, among the layers of transfer that have been learned hierarchically.

(9) $$t_{p},b_{a}=\left(I_{r}^{2}+o_{x}\right)*\sum_{Yi\prime }\left(L_{v}+enc\right)*D_{a}(H_{f})$$

As shown in Equation (9), the pre-trained model and base model are represented as $t_{p}\;and\;b_{a}$, respectively, and the security level is symbolized as $L_{v}$. This model progresses on the encryption and recommendation of handoff $L_{v}\left(enc+H_{f}\right)*D_{a}$. This transfers the data to the nearby vehicle for security level assessments.

Transfer layer and fine-tuning: This defines the vehicle’s existing data and is followed by the next layer containing the pre-trained model. The fine-tuning requires re-training particular information if adversaries or misinterpretations are identified during the exchange. Based on these models, the pre-trained mechanism is evaluated based on the desired handoff and security process.

$$A_{0},U_{g}=\sum_{h_{0}}^{h_{n}}L_{v}+\left(n^{\prime }*x_{h}\right)+\left(k_{p}*dec\right)+D_{a}$$

This is computed as follows:

$$Yi^{\prime }\left(x_{h}\right)=\left(n^{\prime }+n_{v}\right)*\sum_{enc}M_{k}*(A_{1}*p_{t})$$

The formulation is followed up as follows:

(10) $$M_{k}\left(n_{v}\right)=\prod_{A_{1}}D_{a}*\left\{\left(k_{p}-i_{m}\right)+L_{v}\right\},\;for\;all\;D_{a}\in x_{h}$$

As inferred in Equation (10), the transfer layer and fine-tuning works on transfer learning to address the adversary and misinterpretation, which are labelled as $A_{0}\;and\;U_{g}$, respectively. The security level is maintained for the handoff, with recommendations. The next layer is processed using the pre-trained model, where RSU transports the information to the nearby vehicle. The decryption is followed up with the security level for the vehicle-to-vehicle transfer, $L_{v}+\left(n^{\prime }*x_{h}\right)+\left(k_{p}*dec\right)$. The transfer learning evaluates the security level with the handoff process, in which mutual key authentication is followed up. From this evaluation step, the security level analysis of four parameters in transfer learning is completed with the key authentication formulated below.

$$L_{v}=\left(A_{0}+\ldots +A_{n}\right)*\prod_{t_{p}}\left(i_{m}+I_{r}\right)-H_{f},\;for\;all\;H_{f}\in t_{p}$$

This means,

$$M_{k}\left(L_{v}\right)=enc\leftarrow dec\left(A_{0}*p_{t}\right)-k_{p}(\alpha +F_{d})$$

Therefore,

(11) $$F_{d}\left(h_{n},x_{h}\right)=\prod_{A_{0}}^{A_{1}}n^{\prime }\left(enc*pr_{0}\right)*o_{x}-f_{w}$$

**Figure 7:**
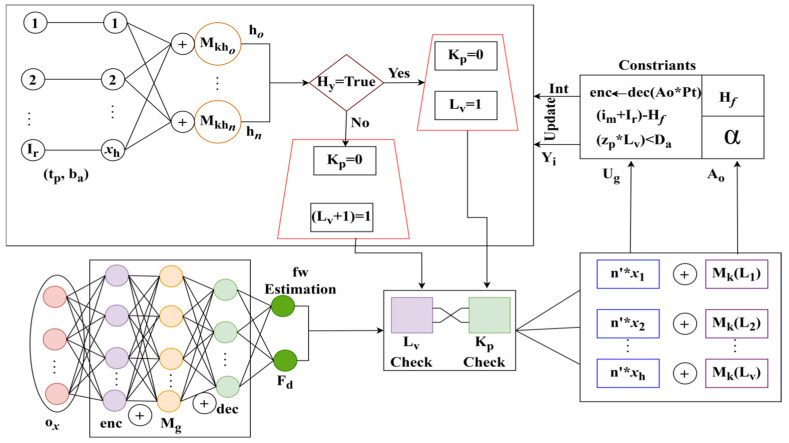
Dodged Transfer Learning Functions for Security Level Estimation.

As found in Equation (11), the security level is maintained using the learning administered using the key authentication with encryption and decryption. Here, $A_{1}$ is the next layer in the transfer process, and, from this case, defines the traffic flow $f_{w}+R^{\prime }\left(z_{p}*L_{v}\right)<D_{a}$. As a condition of the handoff process, a re-handoff is performed if the security level is lower. The dodged transfer learning functions are illustrated in Figure 7.

**Figure 8:**
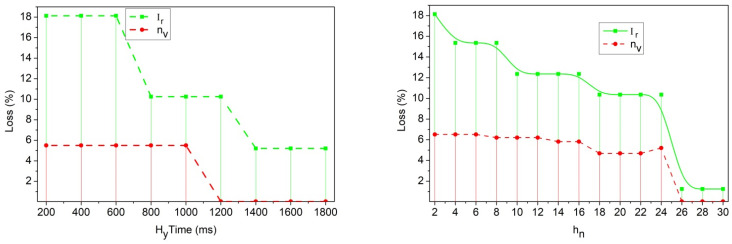
Communication loss assessment for vehicles.

The $\left(t_{b},\;b_{a}\right)$ is the pre-trained model that differentiates $\left(L_{v}=1=L_{v}+1\right)$ for $k_{p}=0$ for $\left(I_{r},x_{h}\right)$ combination. The combination verifies $h_{y}=true$/false to differentiate $L_{v}$ and $\left(L_{v}+1\right)$ for different $o_{x}$ intervals. Then, the transfer layer is consented with, $\left(enc⨁M_{g}⨁dec\right)$, provided that $f_{w}$ or $F_{d}$ is the output. This layer is responsible for $\left(A_{o},U_{g}\right)$ differentiation by inhering $L_{v}$ and $k_{p}$. The tuning for $\left(L_{v}+1\right)$ or $L_{v}$ based on $k_{p}$ using new $H_{y}$ (or) $Y_{i}\left(x_{h}\right)\forall \;enc$ satisfies $H_{y}$ with the same $L_{v}$, based on dec (Figure 7). Based on the above learning, the loss observed during $H_{y}$ for different variants is analyzed in Figure 8.

The communication loss is reduced if the traffic flow is detected using RSU. In this examination step, the misinterpretation is identified and prevents the adversaries from accessing the information $n^{\prime }(o_{x}+x_{h})$. On this basis, communication loss is based on the transportation of information to the vehicle, where the pre-trained model is used to envelope the transfer layer in order to process the secure communication $p_{t}\left(o_{x}*F_{d}\right)+f_{w}$. The loss is addressed, leading to the termination of data exchange if the security degrades, so in this case, communication loss decreases (Figure 8). This addresses the interpretation of traffic-related data communication.

$$R_{f}=H_{f}+\sum_{pr_{0}}\left(A_{1}*x_{h}\right)-dec\left(n^{\prime }-i_{m}\right)+I_{r}\left(o_{x}\right),\;for\;all\;i_{m}\in D_{a}$$

Similarly,

$${=H}_{f}+\sum_{o_{x}}I_{r}*\left(z_{p}+L_{v}\right)\leftarrow dec\left(M_{k}\right)pr_{0}-\left(p_{k}-\alpha \right),\;where\;pr_{0}\in o_{x}$$

Therefore,

(12) $$=\left(o_{x}*pr_{0}\right)+\sum_{z_{p}}L_{v}*\left(i_{m}-k_{p}\right)+D^{\prime }*U_{g},\;for\;all\;U_{g}\in A_{0}$$

**Figure 9:**
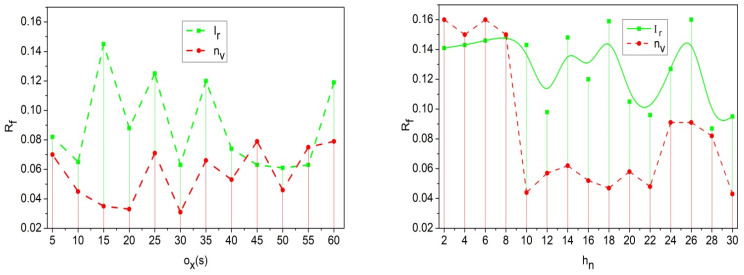
Re-handoff analysis for different vehicles and communication.

As shown in Equation (12), the communication is achieved from one vehicle to another vehicle and follows the transfer layer with fine-tuning, the re-handoff is labelled as $R_{f}$. The process is undertaken with the interaction among the vehicles, where the misinterpretation and congestion are decreased, $\left(i_{m}+\alpha \right)<pr_{0},\;\mathrm{where}\;L_{v}\in R_{f}$. This case defines the following: if the security level is lower, a re-handoff is recommended. Thus, optimization is undertaken for the security level observation with the decryption process, and is expressed as $\left(z_{p}+L_{v}\right)\leftarrow dec\left(M_{k}\right)pr_{0}$. This learning aids the probabilistic detection of security breaches and ensures robust PS for traffic and location information exchange. The PS-based privacy is modified using different handoff authentication levels dictated by mutual lightweight key exchange, reducing adversary control. The $R_{f}$ analysis for different $h_{n}$ and $o_{x}$ is analyzed using Figure 9.

The re-handoff rate decreases for the recommendation method for security level. If the security level decreases, then the handoff takes the role of performing the traffic flow, and the re-handoff then works according to $L_{v}\left(R_{f}\leftarrow H_{f}\right)-R^{\prime }$. This is processed with the recommendation method with which the vehicle navigates the particular road,$\;n_{v}\left(h_{0}\to h_{1}\right)*pr_{0}(M_{g})$. This step illustrates the re-handoff based on the decreased security level and validates the communication with mutual authentication (Figure 9).

## 4. Discussion

The discussion section presents the proposed method comparisons with the existing CACMTM and PMM methods. The metrics computation overhead and security depletion metrics are analyzed in tabulation format. The proposed method is simulated using VANSIM considering 30 vehicles in a road segment with a 10 km distance. This road segment contains five, three, and two intersections for four, three and two crossroads. The road segment comprises five four-leg intersections, three T-intersections, and two two-way crossings. The five four-leg intersections are standard crossroads, where two roads intersect, each having traffic flows from all four directions. The three T-intersections involve one main road intersecting with a side road, forming a ’T’ shape, where traffic from the side road must yield or stop. The two-way crossings are simpler junctions where two roads intersect, but where each road has traffic flowing in only two directions, often requiring fewer traffic control measures. The vehicle density is 30 vehicles per hour, and the speed varies between 20 km/hr and 60 km/hr. A vehicle is allocated 10 to 60 s of interacting time with the RSU, and the maximum RSU deployed in 8 can cover a 500 m radius. The authentication follows a 256-bit encryption key using HECC with temporal characteristics. The minimum handoff time is 180 ms, and the maximum handoff is 320 ms. The re-handoff is to be performed between 60 ms and 210 ms to prevent failure. Based on this simulation setup, the computation overhead for $H_{f}$ and $R_{f}$ is tabulated in Table 2. The variants are vehicle count = 10, 20, and 30 and $o_{x}$ = 20 s, 40 s, and 60 s.

The computation overhead is reduced even as it addresses the congestion and packet losses that lead to the termination of data transportation in smart cities. This illustrates the data exchange process and defines the re-handoff for interaction purposes, $o_{x}\left(n^{\prime }\right)+R_{f}*h_{n}$. The interaction takes place among the vehicles, seeks the traffic flow and defines the mutual key authentication $\left(f_{w}+F_{d}\right)*o_{x}$. The key authentication plays a role in providing access to the secured vehicle in order to reduce the computation overheads (Table 2). Following this analysis, the security depletion of the proposed method is comparatively analyzed with the existing methods, and the values are presented in Table 3. The vehicles range from 5 to 30 for security depletion, and the communication/interaction time is from 10 to 60 s.

The security depletion is less for vehicles where the handoff mechanism works with the knowledge of the congestion and communication losses $L_{v}(\alpha +k_{p})\to h_{1}$. Both the congestion and packet losses are addressed, as is the security. The security level is benchmarked for the vehicle and communicates with nearby vehicles with authentication, $M_{k}(enc(n^{\prime })\to dec(h_{1})$. The authentication relates to the encryption and decryption process and reduces security depletion (Table 3).

Real-time decision-making, the smooth functioning of autonomous vehicles or traffic management systems, and faster data processing are all made possible by a small decrease in computing time for the RPP-PS approach. In the same way, a small security improvement may have a major effect on the system’s resilience, making it less vulnerable to data breaches and other security issues. In addition to improving the user experience and system efficiency, these enhancements might also help ensure the long-term viability of an ITS, which is especially important given the increasing number of connected cars and devices. Although the performance advantages of the suggested solution are quantitatively small, they might significantly affect the overall responsiveness, scalability, and security of ITS systems.

## 5. Conclusions

This article introduced a novel resilient privacy-preserving method with Presumed secrecy for an ITS in smart cities. Presumed secrecy is achieved by reducing re-handoff rates through the maximum possible privacy assessments. Dodged transfer learning is used and the model differs from a conventional ITS process by retaining the previous state until the switchover. After this switchover, mobility, localization, and privacy retention are ensured throughout the driving distance. The lightweight authentication with encryption and decryption concurrency minimizes re-handoffs due to privacy failures. Furthermore, the communication loss for the high interaction intervals defines the security depletion across various dodging processes that maximize the privacy assessment. The proposed method is analyzed using extensive simulation and validated using appropriate parameters and comparative analysis. Presumed secrecy is achieved through privacy assessment. Security administration contains less validation during the intervals. This is observed due to the maximum concurrent access to the roadside units and privacy assessments. Therefore, the assessment interval is less than that of conventional assessment. To address this issue, backwards and forward secrecy with interaction weights will be used and added in the future. This type of interaction improves the privacy level verification across various handoff and re-handoff intervals with unanimous assessments. Compared with existing methods, the proposed approach decreases security depletion rates by 15% across varying traffic densities, underscoring ITS resilience in high-interaction scenarios.

## Figures and Tables

**Table 1:** Summary of the purposes, methodologies and findings from [28,29,30,31,32,33,34,35,36,37].

Author	Purpose	Methodology	Findings
Jiang et al. [28]	It is used to encrypt user’s data from third parties.	BC technology is used here to analyze the keywords during data sharing.	Reduces the computational cost.
Zuo et al. [29]	It is used to ensure the safety and security range of personal data.	Modified Paillier cryptosystem is employed to protect data loss.	Maximizes the performance.
Fan et al. [30]	The model is used to identify the threats that cause issues to PP policies.	A decentralized autonomous organization (DAO) is implemented in the model to analyze the relevant data for providing PP services.	Elevates the effectiveness level of the systems.
Zhu et al. [31]	It provides feasible security policies to ensure the safety of users’ data.	The reinforcement learning (RL) algorithm is also used here to encrypt the necessary data before sharing processes.	Reduces the communication cost and application overheads.
Gupta et al. [32]	It is used to enhance the performance of communication services.	ML algorithm is used here to detect unwanted threats while travelling.	Elevates the efficiency range of the systems.
Zhang et al. [33]	It is used to ensure the safety and privacy level of the communication process.	V2C requires effective PP policies to secure data while sharing.	Increases the performance range of the system.
Sun et al. [34]	It is used to analyze the main source of the images.	The actual purpose and necessity of the images are identified for the encryption process.	Minimizes the computational cost and complexity range.
Sun et al. [35]	It is used to enhance the storage capacity by improving the security.	BC technique is employed here to identify the optimal authentication and access request over data.	Enhances the efficiency and performance range of the systems
Xu et al. [36]	It is used to solve privacy issues while performing communication services.	BC is used to analyze the key values and factors for the authentication process.	Increases the effectiveness ratio.
Qiong Wu et al. [37]	It provides edge caching for next-generation networks by empowering caching units in small-cell base stations (SBSs), allowing user equipment (UE) to fetch users’ requested contents that are pre-cached in an SBS.	A cooperative edge caching scheme based on elastic federated and multi-agent deep reinforcement learning (CEFMR) is used to optimize the cost in the network.	Enhancing the overall performance ratio

**Table 2:** Computation overhead assessment.

	$H_{f}$ Overhead			$R_{f}$ Overhead		
Vehicles	10	20	30	10	20	30
CACMTM [25]	45.25	48.36	51.06	38.69	37.36	39.47
PMM [26]	41.56	45.36	42.36	32.14	38.69	32.36
RPP-PS	25.485	25.361	26.472	22.474	24.365	27.541
$o_{x}$	**20 s**	**40 s**	**60 s**	**20 s**	**40 s**	**60 s**
CACMTM [25]	48.69	48.36	51.04	48.25	45.36	47.47
PMM [26]	32.145	35.69	39.6	29.36	31.23	28.78
RPP-PS	24.892	24.987	25.478	27.417	24.369	25.496

**Table 3:** Comparative assessment of security depletion.

	Depletion					
Vehicles	5	10	15	20	25	30
CACMTM [25]	0.1558	0.1574	0.1589	0.1574	0.1561	0.1548
PMM [26]	0.1547	0.1536	0.1525	0.1564	0.1512	0.1498
RPP-PS	0.1467	0.1485	0.1477	0.1487	0.1489	0.1491
$o_{x}$	**10 s**	**20 s**	**30 s**	**40 s**	**50 s**	**60 s**
CACMTM [25]	0.1587	0.1571	0.1569	0.1517	0.1523	0.1544
PMM [26]	0.1541	0.1532	0.1521	0.1518	0.1511	0.1489
RPP-PS	0.1471	0.1463	0.1481	0.1496	0.1478	0.1467

## Data Availability

The data will be made available by the authors on request.
